# Hypercoagulability and Inflammatory Markers in a Case of Congenital Thrombotic Thrombocytopenic Purpura Complicated by Fetal Demise

**DOI:** 10.3390/jcm11237115

**Published:** 2022-11-30

**Authors:** Leslie Skeith, Kelle Hurd, Shruti Chaturvedi, Lorraine Chow, Joshua Nicholas, Adrienne Lee, Daniel Young, Dawn Goodyear, Jennifer Soucie, Louis Girard, Antoine Dufour, Ejaife O. Agbani

**Affiliations:** 1 Department of Family Medicine, Cumming School of Medicine, University of Calgary, Calgary, AB T2N 4N1, Canada; 2 Department of Medicine, Johns Hopkins University School of Medicine, Baltimore, MD 21205, USA; 3 Department of Anaesthesiology, Perioperative and Pain Medicine, Cumming School of Medicine, University of Calgary, Calgary, AB T2N 4N1, Canada; 4 Department of Medicine, University of British Columbia, Vancouver, BC V6T 1Z4, Canada; 5 McCaig Institute for Bone and Joint Health, University of Calgary, Calgary, AB T2N 4N1, Canada; 6 Department of Obstetrics and Gynecology, Cumming School of Medicine, University of Calgary, Calgary, AB T2N 4N1, Canada; 7 Department of Physiology & Pharmacology, Cumming School of Medicine, University of Calgary, Calgary, AB T2N 4N1, Canada; 8 Libin Cardiovascular Institute, University of Calgary, Calgary, AB T2N 1N4, Canada

**Keywords:** platelets, thrombosis, procoagulant membrane dynamics, inflammatory markers, congenital, thrombotic thrombocytopenic purpura, fetal demise

## Abstract

Background: Congenital thrombotic thrombocytopenic purpura (cTTP) is a rare disorder caused by an inherited genetic deficiency of ADAMTS13 and affects less than one per million individuals. Patients who are diagnosed with TTP during pregnancy are at increased risk of maternal and fetal complications including fetal demise. We present a case of a 32-year-old G3P0 (gravida 3, para 0) who presented at 20 weeks gestation with a new diagnosis of congenital TTP (cTTP) and fetal demise. Methods: We describe the pathophysiology of pregnancy complications in a patient with cTTP using platelet procoagulant membrane dynamics analysis and quantitative proteomic studies, compared to four pregnant patients with gestational hypertension, four pregnant patients with preeclampsia, and four healthy pregnant controls. Results: The cTTP patient had increased P-selectin, tissue factor expression, annexin-V binding on platelets and neutrophils, and localized thrombin generation, suggestive of hypercoagulability. Among 15 proteins that were upregulated, S100A8 and S100A9 were distinctly overexpressed. Conclusions: There is platelet-neutrophil activation and interaction, platelet hypercoagulability, and proinflammation in our case of cTTP with fetal demise.

## 1. Introduction

Patients who are diagnosed with thrombotic thrombocytopenic purpura (TTP) during pregnancy are at increased risk of fetal and maternal complications. There is a high rate of preterm birth, preeclampsia, late fetal demise including stillbirth, and maternal complications such as neurological, renal, or arterial sequelae, and rarely maternal death [1,2,3,4,5,6]. Thrombotic thrombocytopenic purpura may occur for the first time in pregnancy or the postpartum period and may be acquired (autoimmune) or congenital [7]. Fetal complications are more likely to occur in the first pregnancy and/or when the patient is not receiving prophylactic therapy [1]. Here, we present a case of congenital TTP (cTTP) during pregnancy (ADAMTS13 activity < 10% and *ADAMTS13* gene variants) that was complicated by fetal demise. Congenital TTP is a form of a thrombotic microangiopathy where a severe deficiency of ADAMTS13 due to *ADAMTS13* gene mutations leads to ultra-large VWF multimers accumulating, which binds to endothelial surfaces and platelets to promote microvascular thrombosis [8]. In a small case series of pregnant patients with cTTP who had pregnancy complications, there was macroscopic evidence of fetal and maternal vascular lesions of under perfusion in their placentas, along with intraplacental infarcts, fibrin thrombi, and intervillous fibrin deposition [9]. To understand the pathophysiology further, we evaluated platelets, neutrophils, and inflammatory markers to elucidate the pathophysiology of complications associated with cTTP in pregnancy. Our platelet imaging, immunofluorescence, and proteomics studies identified interactions on a molecular level in a case of cTTP in pregnancy.

## 2. Case

We report a case of a 32-year-old G3P0 (gravida 3, para 0) who presented at 20 weeks gestational age (GA) with new cTTP and fetal demise. The patient had an elevated body mass index (BMI 38 during pregnancy) with no other medical conditions and had spontaneous abortions at 6 weeks and 9 weeks GA of unknown cause, which included normal cytogenetics of the fetuses. The maternal platelet counts at the time of the earlier pregnancy losses were unknown. In addition to a prenatal vitamin including folate, the patient was started on aspirin 81 mg daily during her most recent pregnancy to prevent placental-mediated complications. She had a normal platelet count in first trimester (338 × 10^9^/L) and normal obstetric ultrasounds (12^5^ weeks GA; 19^4^ weeks GA). The patient presented at 19^5^ weeks GA with epigastric pain and intermittent high blood pressure (range 130/70 to 186/96), with a platelet count 194 × 10^9^/L, LDH 306 U/L (Upper limit of normal, ULN 100 U/L), ALT 41 U/L (ULN 39 U/L), normal urate, creatinine 49 µmol/L, and no proteinuria. She presented to hospital again at 20^3^ weeks GA with petechiae, hyperreflexia, a platelet count of 13 × 10^9^/L, microangiopathic hemolytic anemia (hemoglobin 95 g/L, LDH 833 U/L, haptoglobin < 0.15 g/L, reticulocyte count 5.3%, bilirubin 19 µmol/L [ULN 24 µmol/L]), and occasional schistocytes. She also had an elevated ALT 42 U/L, elevated labile blood pressure, creatinine 95 µmol/L (approximately doubled), and new proteinuria of 0.162 g/mmol. Although fetal viability was confirmed upon initial presentation, an intrauterine fetal death occurred during the following twelve hours. The placenta showed marked increased intervillous fibrin scattered with acute intervillositis, decidual necrosis, and hemorrhage. Chromosome microarray analysis and karyotyping of the fetus was normal. 

While the working diagnosis was preeclampsia/HELLP (Hemolysis, Elevated Liver Enzymes, Low Platelets) syndrome, the critically low platelets also kept TTP on the differential. Within 24 h of presentation, ADAMTS13 activity testing confirmed TTP. Using ADAMTS13 activity and inhibitor profile tests, a diagnosis of cTTP was made based on severely deficient ADAMTS13 activity of 0.69% (normal 40–130%) and no ADAMTS13 antibodies (<12 units/mL), and two heterozygous *ADAMTS13* variants; with *ADAMTS13* c.578G>A, p.(Arg193Gln), a missense variant previously reported in cTTP [10] and *ADAMTS13* variant c.2420+4_2420+19del (Blueprint Genetics, Seattle, WA, USA). The second variant is in the intronic splice region and has not previously been described. Autoimmune testing including ANA, ENA, ds-DNA, C3, C4, C50, and antiphospholipid antibodies (anticardiolipin antibody, anti-beta-2 glycoprotein antibody, and lupus anticoagulant) were negative. 

Once the ADAMTS13 activity result returned, the patient received a plasma infusion and high-dose steroids, followed by an induction of labor and 6 cycles of plasma exchange. Her blood pressure, renal function, platelet count, and hemolytic markers normalized by postpartum day 5 without evidence of recurrent thrombocytopenia or hemolysis, although her anemia took a month to fully resolve. Outside of this episode, her ADAMTS13 activity remained <10% and ADAMTS13 antibody testing remained negative, with results available up to 4 months postpartum. 

## 3. Materials and Methods

**Figure 1:**
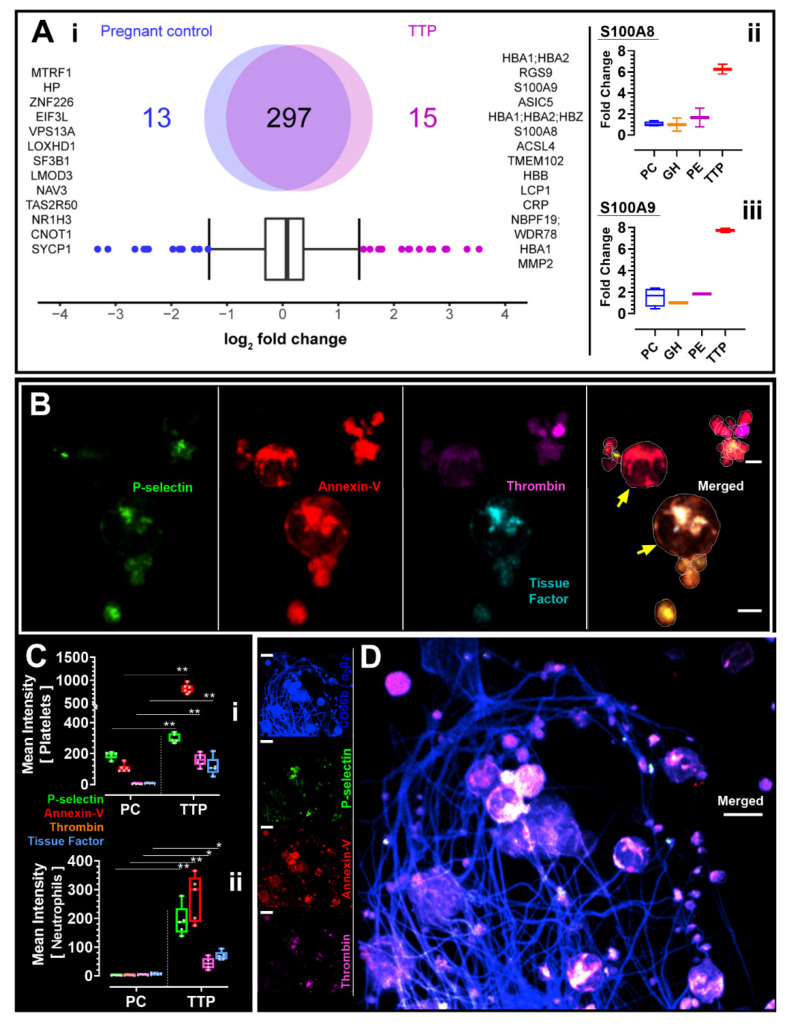
Platelet Proteomics and Procoagulant Membrane Dynamics Study in a Case of Congenital TTP Complicated by Fetal Demise.

We completed platelet procoagulant membrane dynamics analysis and quantitative proteomic studies in a patient with cTTP, as well as control participants. Study participants were screened and enrolled between July and December 2021. Control participants, but not the cTTP participant, were enrolled in a related preeclampsia study [11]. We utilized a detailed fluorescent imaging approach which we previously described [12,13], in platelet-rich-plasma re-constituted to contain neutrophils (PRP+). We derived PRP+ fractions by centrifuging whole blood at 180× *g* for 17 min, followed by a careful extraction of both the upper plasma-platelet fraction (PRP) and the buffy coat [14]. Citrated PRP+ from study participants were allowed to adhere to bovine serum albumin coated surfaces. Extended focus images at the 45 min timepoint are shown. To visualize homotypical and heterotypical platelet microaggregate or microthrombi in whole plasma (Figure 1B,D), we examined PRP+ fractions. Platelets were labelled with Alexa-fluor^®^ 488 anti-human CD62P (P-Selectin) antibody to detect membrane P-selectin exposure, Alexa-fluor^®^ 568 Annexin-V to monitor phosphatidylserine (PS) externalization, and Alexa-fluor^®^ 405 anti-human tissue factor antibody. In addition, Alexa-fluor^®^ 647 conjugated mouse monoclonal antibody specific for an epitope, mapped between amino acids 331–376 within an internal region of human thrombin, was used to determine platelet membrane thrombin generation. We then conducted plasma quantitative shotgun proteomics analysis as previously reported [11], and compared proteomics and platelet imaging in the described patient with cTTP to 4 healthy pregnant controls (PC), 4 gestational hypertension (GH), and 4 preeclampsia (PE) patients (Research Ethics Board Approval #REB18-1545). The proteomics data of our study are publicly available via ProteomeXchange with identifier PXD037898. The R codes are available upon request. Additional data relating to healthy PCs, GH, and PE participants have been reported elsewhere [11]. Study data were analyzed using GraphPad Prism 9.3 (Dotmatics, San Diego, CA, USA). Statistical significance was determined by 2-way analysis of variance (ANOVA) test, followed by Sidak multiple comparison tests; *p* < 0.05 (*) or *p* < 0.01 (**) were considered significant. 

## 4. Results

In addition to a pregnant patient with cTTP, samples were also drawn from four PCs (mean age 32, mean GA 39.4 weeks, range 49–40.2 weeks), four GH (mean age 35, mean GA 35.8 weeks, range 32–40.8 weeks), and four preeclampsia patients (mean age 32, GA 31.4 weeks, range 24.6–36.6 weeks) within a six-month period; all control participants had a normal hemoglobin, platelet count, and creatinine values.

We identified 15 proteins upregulated in TTP (Figure 1(A-i)), and sub-analysis revealed that S100A8 and S100A9 were distinctly overexpressed (6-7-fold increase) in our TTP patient, but not in the controls used in this study with PC, GH, or PE. (Figure 1(A-ii,iii)). High-resolution platelet imaging from our TTP patient showed acquired platelet and neutrophil activation (determined through signals of fluorescent indicators) and classic structures of platelet-neutrophil aggregates [15,16] (Figure 1B). Compared to all other participant groups, our cTTP patient had increased P-selectin (mean ± SD, PC vs cTTP: 190.2 ± 23.5 vs 299.6 ± 29.1; *p* = 0.0033), tissue factor expression (mean ± SD, PC vs cTTP: 5.6 ± 3.5 vs 116.2 ± 60.4; *p* = 0.0029), annexin-V binding (mean ± SD, PC vs cTTP: 105.5 ± 24.9 vs 819.5 ± 99.3; *p* < 0.001), and localized thrombin generation on platelets (mean ± SD, PC vs cTTP: 3.5 ± 3.2 vs 159.7 ± 40.1; *p* < 0.001), suggestive of hypercoagulability (Figure 1(C-i)). Results were similar in neutrophil analysis (Figure 1(C-i)). 

We visualised in the plasma sample of the described participant with cTTP, but not in the PC, GH, or PE control samples, loose thrombus consisting of activated and procoagulant platelets and neutrophils trapped within mesh-like structures resembling a fibrin-network, as measured by increased staining of procoagulation markers (Figure 1D). The range of procoagulation signals from the cells of the one cTTP participant is relatively larger compared to measures from control PCs. Likely this is indicative of the relatively quiescent/non active state of platelets and neutrophils in PC compared to cTTP where active procoagulation is progressing at varying degrees in the different cell population analysed in cTTP. This notwithstanding, the minimum, mean, median, and maximum procoagulation values recoded were higher in cTTP, compared to PCs.

Quantification results of plasma proteomics of pregnant controls vs. one patient with thrombotic thrombocytopenic purpura (TTP) as illustrated by Venn Diagrams (Figure 1(A-i)). Interquartile box plot analysis was performed to identify the differently expressed proteins as represented by outliers. “(Figure 1(A-ii)) S100A8 and (Figure 1(A-iii)) S100A9 intensities between pregnant control (PC), gestational hypertension (GH), preeclampsia (PE), and congenital TTP (TTP) as quantified by shotgun proteomics. Data are represented as boxplots. (Figure 1B,D): Images shown in B and D were from the one patient with cTTP. Yellow arrows in B are pointing to procoagulant neutrophils interacting with activated platelets. In (Figure 1C), fluorescent signal intensity data from the 4 PC participants and the one TTP patient (replicates shown) were analyzed using GraphPad Prism 9.3 (Dotmatics, San Diego, CA, USA) and presented as box-and-whiskers plots showing minimum to maximum values, replicate data inclusive. Images were captured at Nyquist using Nikon A1R laser scanning confocal microscope (original objective magnification, ×60) and analyzed using Volocity^®^ Software (Quorum Technologies, Laughton, UK). Scale bars: 3 μm (Figure 1B), 7 μm (Figure 1D).

## 5. Discussion

Thrombocytopenia and microangiopathic hemolytic anemia in pregnancy are manifestations of HELLP syndrome, preeclampsia, TTP, antiphospholipid syndrome, all of which have overlapping clinical and biochemical features that can make it difficult to distinguish between them [17]. Congenital TTP is a rare disorder caused by an inherited genetic deficiency of ADAMTS13 and affects less than one per million individuals, and makes up <5% of all TTP cases [8]. TTP can present for the first time in pregnancy or the postpartum period, and up to 25% of pregnancy-associated TTP cases are from cTTP [1]. In this case, cTTP was confirmed by ADAMTS13 activity < 10%, negative ADAMTS13 antibody testing, and biallelic mutations with at least one mutation previously reported with cTTP [10]. Furthermore, the ADAMTS13 level remained low (<10%) beyond the 3-month postpartum period with no ADAMTS13 antibodies detected, further supporting the diagnosis of cTTP. 

There is overlap between TTP and preeclampsia. While TTP has been reported more often in the third trimester and postpartum, fetal loss may be highest in the second trimester [4]. Another observational study identified TTP presentations more commonly occurring in the second trimester and early postpartum [5]. Preeclampsia, typically diagnosed after 20 weeks gestation, is a common feature of pregnancies complicated by TTP, so proteinuria and a clinical overlap may be seen [5,6,18]. This supports the use of low-dose aspirin for preeclampsia prevention in pregnant patients with known TTP. While not specific to this case, ADAMTS13 levels are lower in preeclampsia in the absence of TTP, although are not as severe (defined as <10%) and is an area of future research in preeclampsia and other conditions such as antiphospholipid syndrome [19,20,21].

While the condition is rare, delays in the diagnosis of TTP in pregnancy can lead to important maternal and fetal complications. In addition to the diagnostic challenge of having clinical overlap with other conditions, the turnaround time of the ADAMTS13 activity testing is variable and is often not available to make immediate decisions. We advocate for pursuing early investigations for TTP, and to initiate empiric therapies while test results are pending, depending on the clinical scenario.

Platelets have a proinflammatory role; using P-selectin and beta(2) and beta(3) integrins (CD11b/CD18, CD41/CD61), platelets can interact with neutrophils to promote the recruitment of neutrophils into inflammatory tissue [22]. Furthermore, platelet–neutrophil interaction has been reported in other prothrombotic conditions such as in severe SARS-Cov-2 infection, thrombosis, atherosclerosis, and tissue injury and repair [23]. Our data are hypothesis generating on the role of platelets and inflammation in the area of cTTP-related pregnancy complications.

There are limitations to this study evaluating platelet, neutrophils, and inflammatory markers. This study is a single case report of cTTP and there are small sample sizes of control participants. We were not able to control for other factors in the control participants, such as age, BMI, gestational age, or prior pregnancy outcomes. Additionally, we are not able to describe temporal changes in a pregnancy with only a single time point drawn. We chose a control group that was at risk of similar placental complications, in hopes to better understand the pathophysiology of placental complications related to cTTP. However, this group was not exactly matched because they did not have low platelets and microangiopathic hemolytic anemia or had an outcome of a fetal demise. The inflammation seen in the participant with cTTP could have been the body’s inflammatory response to a fetal demise and not the preceding cause. Lastly, our research testing methods are not available for routine use in practice, so how to implement additional testing into clinical practice deserves further study. Further research is still needed to understand the implications of our findings.

## 6. Conclusions

This case report highlights the challenging diagnostic overlap with other clinical TTP syndromes like preeclampsia/HELLP syndrome, and that additional laboratory ADAMTS13 testing can be invaluable. Using platelet procoagulant membrane dynamics and proteomics studies, we identified platelet–neutrophil activation and interaction, platelet hypercoagulability, and proinflammation in the case of cTTP with a fetal demise. However, further research is still needed to confirm our findings and the pathophysiology of pregnancy complications of fetal demise and preeclampsia in pregnant patients with TTP.

## Data Availability

The proteomics data of our study are publicly available via ProteomeXchange with identifier PXD037898. The R codes are available upon request.
